# Interactions between the tumor microbiota and breast cancer

**DOI:** 10.3389/fcimb.2024.1499203

**Published:** 2025-01-24

**Authors:** Hua Guo

**Affiliations:** The Nursing Department, Shaanxi Provincial People’s Hospital, Xi’an, Shaanxi, China

**Keywords:** breast cancer, tumor microbiome, intestinal microbiota, intratumoral microbiota, microbial metabolites

## Abstract

Breast cancer is the most common malignancy in women worldwide. Changes in the microbiota and their metabolites affect the occurrence and development of breast cancer; however, the specific mechanisms are not clear. Gut microbes and their metabolites influence the development of breast cancer by regulating the tumor immune response, estrogen metabolism, chemotherapy, and immunotherapy effects. It was previously thought that there were no microorganisms in breast tissue, but it is now thought that there are microorganisms in breast cancer that can affect the outcome of the disease. This review builds on existing research to comprehensively analyze the role of gut and intratumoral microbiota and their metabolites in the development and metastasis of breast cancer. We also explore the potential function of the microbiota as biomarkers for prognosis and therapeutic response, highlighting the need for further research to clarify the causal relationship between the microbiota and breast cancer. We hope to provide new ideas and directions for the development of new methods for breast cancer treatment.

## 1 Introduction

Global cancer statistics for 2022 indicate that breast cancer will be the most commonly diagnosed cancer among women, with an estimated 2.3 million new cases every year, representing 11.6% of all cancer cases (Bray et al., 2024). With the continuous increase in research on breast cancer and the tumor microenvironment, treatment methods for the disease have become increasingly targeted, typically involving a combination of traditional therapy and novel immune therapy guided by the cancer molecular subtype. Although treatment regimens for breast cancer have been continuously optimized, the therapeutic effect for highly malignant breast cancer is still not ideal, with challenges such as drug resistance, recurrence, and distant metastasis. Therefore, identifying new treatment directions is helpful for the clinical selection of more effective treatment plans.

The large microbiota, composed of bacteria, viruses, and eukaryotes that inhabit the human body, play an important role in maintaining health and disease development. In a healthy human body, the microbiota coexists peacefully with the organism and assists in maintaining health. However, when the composition of the microbiota is unbalanced, diseases, including tumors, may occur (Lynch and Pedersen, 2016; Fan and Pedersen, 2021). These microbial organisms indirectly influence cancer through mechanisms, such as metabolite production and immune system modulation, which affect both distant and proximal tumor tissues (Yang et al., 2023a). Currently, research on tumor-related microorganisms mainly focuses on intestinal microorganisms. These microorganisms and their metabolites are crucial for maintaining the integrity of the intestinal mucosa, nutrient metabolism, immune regulation, and other functions (Li S. et al., 2024). This is particularly evident in colorectal cancer (CRC), where the gut microbiota directly affects the tumor microenvironment (TME) by regulating the immune system, thereby influencing CRC prognosis (Garrett, 2015; Xavier et al., 2020).

Relatively few studies have addressed the involvement of microbiota in breast cancer progression, particularly the impact of intratumoral microorganisms. Significant alterations in the breast microbiota have been detected in patients with malignancy, with notable differences between cancerous tissues and healthy controls, and between benign and malignant breast tissues (Bobin-Dubigeon et al., 2021; Ma et al., 2022). Microbial dysbiosis in other organs may also contribute to breast cancer development. For example, oral dysbiosis-mediated periodontal disease is involved in the development of breast cancer (Jia et al., 2020; Zheng et al., 2022). This indicates that both intratumoral microorganisms and microorganisms in other parts of the body can affect the progression of breast cancer.

This study systematically summarizes the roles of intestinal and intratumoral microorganisms in the development of breast cancer and seeks new ideas for the prevention and treatment of breast cancer.

## 2 Detection methods for tumor microorganisms

Advancements in high-throughput sequencing technology have continuously improved the sequencing accuracy of microorganisms while reducing costs. 16S rRNA and metagenomic sequencing are the most widely employed approaches for detecting the distribution and characteristics of microorganisms in patients (Clarridge, 2004; Goodrich et al., 2014; Knight et al., 2018; Liu et al., 2021b). The 16S rRNA gene, which is common to both bacteria and archaea, consists of nine variable regions (V1-V9) and 10 conserved regions arranged alternately. Conserved regions facilitate primer design for gene amplification, whereas variable regions reflect evolutionary differences between species, making the 16S rRNA gene a widely used molecular marker for prokaryotic identification, classification, phylogenetic analysis, and diversity studies.

Compared to 16S rRNA gene sequences, metagenomics provides a broader spectrum of microbial information. An effective approach in metagenomics is the recovery of metagenome-assembled genomes (MAGs), which allow the reconstruction of microbial genomes from metagenomic data (Zhou et al., 2022). Additionally, novel microbiome analysis methods, such as machine learning-based multiomics analysis, have shown promise in predicting the characteristics of the human microbiome related to complex host diseases (Asnicar et al., 2024).

Other sensitive microbial detection techniques, including immunohistochemistry (IHC), fluorescence *in situ* hybridization (FISH), D-alanine labeling, and tissue isolation culture, can also be used to detect intratumoral bacterial biomass (Xue et al., 2023). IHC detects bacterial samples using antibodies against bacteria-derived lipopolysaccharides (LPS) or lipoteichoic acid (LTA). FISH can identify bacterial DNA in tissues using fluorescent dye-labeled probes that target the 16S rRNA gene (Lin et al., 2021). Furthermore, most bacteria exhibit alanine racemase activity, which is essential for D-alanine biosynthesis and peptidoglycan formation in bacterial cell walls, making D-alanine labeling a useful method for detecting live bacteria *in situ*.

## 3 Influence of the intestinal microbiota on breast cancer

Alterations in the intestinal microbial diversity can lead to intestinal microecological dysbiosis, thereby promoting the onset of various diseases (Sekirov et al., 2010). Intestinal microecological dysbiosis can affect the prognosis of diseases, including tumors, through multiple mechanisms, such as activating innate immune responses, pro-inflammatory responses, changes in metabolites, regulation of estrogen levels, and alteration of drug metabolism (Goedert et al., 2018; Arnone and Cook, 2022; Niekamp and Kim, 2023; Wang et al., 2023; Bernardo et al., 2023a; Giampazolias et al., 2024). Compared with healthy people, the diversity of intestinal microorganisms in patients with breast cancer is reduced, characterized by a depletion of *Bacteroidetes*, *Odoribacter*, *Butyricimonas*, and *Coprococcus* and an enrichment of *Firmicutes*, *Acidaminococcus*, *Tyzzerella*, *Hungatella, Porphyromonas*, and *Peptoniphilus* (Bobin-Dubigeon et al., 2021; Ma et al., 2022; Song et al., 2022; Altinok Dindar et al., 2023; Amaro-da-Cruz et al., 2024). These studies indicated that intestinal microecological dysbiosis may affect the occurrence, development, and therapeutic effects of breast cancer through multiple pathways.

### 3.1 Intestinal microbiota regulates the immune response of breast cancer

Alterations in anti-tumor immune responses and chronic inflammation are important factors influencing the occurrence of various tumors. Microbial dysbiosis (gut microbiota that express the enzyme β-glucuronidase), characterized by chronic inflammation and immune evasion, can promote tumorigenesis in breast cancer (Buchta Rosean et al., 2019; Arnone and Cook, 2022). Gut microorganisms (e.g. *Proteobacteria and Firmicutes*) can modulate the immune system through regulating lymphocyte proliferation via bacterial metabolites and influencing chronic inflammation and estrogen metabolism (Van der Merwe et al., 2021; Zhang et al., 2021). Furthermore, commensal bacteria (e.g. *Helicobacter hepaticus*) also promotes the occurrence and distant metastasis of breast cancer by influencing the anti-tumor immune functions of IL-6 and neutrophils in the tumor microenvironment (Lakritz et al., 2015; Rutkowski et al., 2015). These studies indicate that immune system disorders induced by intestinal microecological dysbiosis play an important role in the development of breast cancer.

Toll-like receptors (TLRs) are essential components of innate immune responses. They serve as pattern recognition receptors (PRRs) that detect various pathogens, including commensal microbiota. TLR4/MyD88 stimulation by *Fusobacterium nucleatum (F. nucleatum)* promotes tumor development via NF-κB activation in CRC (Yang et al., 2017; Yu et al., 2017; Zhang et al., 2019). Similar TLR4 and NF-κB activation has been reported in breast cancer cells stimulated by bacterial LPS, leading to the expression of inflammatory factors and apoptotic proteins (Rajput et al., 2013). Moreover, microorganisms can drive malignant progression at extra-mucosal sites via TLR5-dependent signals to increase the expression of systemic IL-6 and immunosuppressive γδ T cells to regulate tumor-promoting inflammation (Rutkowski et al., 2015). These studies indicate that intestinal microorganisms and their metabolites promote the growth and metastasis of breast cancer cells by regulating TLR-mediated innate immune responses.

### 3.2 Intestinal microbiota affects estrogen metabolism

Estrogen exposure is an important factor that affects the occurrence and development of breast cancer. The “estrobolome” refers to the collection of intestinal bacterial genes whose metabolites can metabolize estrogen (Plottel and Blaser, 2011). Estrogen is mainly produced by the ovaries, adrenal glands, and adipose tissues, circulates in the blood in the form of free or conjugated estrogen, and combines with its metabolites in the liver to form conjugated estrogen. Conjugated estrogen is metabolized into water-soluble molecules and excreted in urine or bile. The conjugated estrogen in bile can be decomposed by bacteria with β-glucuronidase activity in the intestine, and after reabsorption and re-entry into the circulation, it increases the bioavailability of estrogen. Circulating estrogen stimulates growth and proliferation of breast cells (Kwa et al., 2016). Therefore, the enterohepatic circulation of estrogen can affect the levels of estrogen and its metabolites in the circulatory system and may ultimately contribute to the risk of hormone-driven breast cancer.

The process by which the intestinal “estrobolome” regulates the enterohepatic circulation and reabsorption of estrogen is also influenced by host factors, such as age, diet, and antibiotics. The effects of antibiotics and diet are discussed below.

#### 3.2.1 Antibiotics

The use of antibiotics can affect the diversity and quantity of the flora in the body. Improper use can lead to dysbiosis of the intestinal flora and various diseases, including breast cancer (Velicer et al., 2004; Garcia Rodriguez and Gonzalez-Perez, 2005; Sorensen et al., 2005; Friedman et al., 2006). Ampicillin and oxytetracycline can increase the content of conjugated estrogen in the feces of women and men, respectively, while reducing estrogen in the urine (Adlercreutz et al., 1975; Martin et al., 1975; Hamalainen et al., 1987). Increased antibiotic exposure may also increase the risk of breast cancer (Velicer et al., 2004; Friedman et al., 2006). These studies indicate that certain antibiotics ultimately affect the risk of breast cancer by regulating estrogen excretion and influencing the deconjugative activity of intestinal bacteria. However, the mechanism by which antibiotics affect the development of breast cancer through the intestinal flora remains unclear and requires further research.

#### 3.2.2 Diet

Although factors such as lifestyle, exercise, and supplements can affect estrogen levels, diet remains a major factor influencing the overall estrogen concentration, potentially through the modulation of gut microbiome composition and function (Muegge et al., 2011). As early as 1982, Goldin et al. found that vegetarians excreted higher levels of conjugated estrogens in feces than non-vegetarians, resulting in lower plasma estrogen levels (Goldin et al., 1982).

Adiposity has been linked to higher serum estrogen levels in postmenopausal women, which are correlated with an increased risk of multiple malignancies (Keum et al., 2015). High-fat diet (HFD) disrupts gastrointestinal metabolism and immune homeostasis and contributes to disease states. Soto-Pantoja et al. found that HFD mice and mice that received fecal transplantation from HFD-fed mice exhibited an increased Firmicutes/Bacteroidetes (F/B) ratio (Soto-Pantoja et al., 2021). Microbiota changes observed in genetically obese mice are consistent with those observed in obese humans (Ley et al., 2005, 2006). This altered ratio increases the abundance of harmful bacteria, leading to the release of enterotoxins and chronic low-grade inflammation (Mikó et al., 2019; Parida and Sharma, 2019). The HFD promotes cancer progression by inducing gut microbiota-mediated leucine production and polymorphonuclear myeloid-derived suppressor cell differentiation (Chen et al., 2024b). The above studies indicate that the gut-breast signaling axis is involved in regulating the influence of diet on breast cancer risk, which provides a reference for guiding the daily dietary intake of breast cancer patients and women at a high risk of breast cancer.

### 3.3 Effects of intestinal microbiota on breast cancer treatment

The gut microbiome plays a significant regulatory role in modulating responses to both traditional and immune therapies (Battaglia et al., 2024). They can regulate local inflammation and gut barrier function by targeting drug metabolism, modulating immune responses, and secreting different metabolites, ultimately affecting chemotherapy outcomes (Alexander et al., 2017; Sampsell et al., 2020). Chemotherapy drugs may also affect chemotherapy-induced weight gain and neurological side effects by regulating microbial diversity (Terrisse et al., 2021).

HER2 inhibitors such as trastuzumab have a good therapeutic effect on HER2-positive breast cancer. However, antibiotic treatment changes the composition of the intestinal flora, reduces dendritic cell activation and IL12p70 secretion, leads to changes in anti-tumor immunity in the tumor microenvironment, and ultimately reduces the therapeutic activity of trastuzumab (Di Modica et al., 2021). This study indicates that intestinal microbial dysregulation can regulate the host immune system and ultimately affect the therapeutic effects of chemotherapeutic drugs.

The immune checkpoint blockade (ICB) is a new-generation immunotherapeutic strategy for various cancers. Commensal gut bacteria can suppress inflammation, reshape primary and acquired immune responses, and reprogram the TME in murine models and patients, thereby influencing ICB efficacy (Xue et al., 2024). Jia et al. reported that the gut microbial metabolite indolepropionic acid (IPA) enhances immunotherapy efficacy by modulating T cell stemness in cancers (Jia et al., 2024). Additionally, using a murine model of gut microbiota dysbiosis, Shi et al. found that *Lactobacillus* and its metabolite lactic acid promote breast cancer progression, particularly triple-negative breast cancer (TNBC), by affecting the anti-tumor activities of immune cells in the TME (Shi et al., 2023). These results indicate that intestinal microbiota is expected to become a potential target for breast cancer treatment.

### 3.4 Microbial metabolites affect tumor immune microenvironment and therapeutic response in breast cancer

A large part of the physiological regulatory function of microorganisms is exerted through their metabolites such as short-chain fatty acids (SCFAs), bile acids, and inosine. These metabolites enter blood circulation, serve as significant modulators of the TME, and influence immune cell differentiation signals and the release of substances from both immune cells and tumors (Jaye et al., 2022a; Yang et al., 2023b).

#### 3.4.1 Short-chain fatty acids

SCFAs, primarily butyrate, propionate, and acetate, are produced by microbiotal dietary fiber digestion. They are tumor suppressors in various cancer types, particularly colon cancer. SCFAs are the most common gut microbial metabolites and are mainly produced by intestine-colonizing species, such as *Eubacterium rectale*, *Clostridium leptum*, and *Faecalibacterium prausnitzii* (Williams et al., 2017; Jaye et al., 2022b). The total concentration of SCFAs exceeding 100 mM in the intestine include propionate, acetate, and butyrate (Mirzaei et al., 2021). Butyrate, one of the most abundant SCFAs, has a dual effect on cancer cell proliferation, which is largely dependent on its concentration; low concentrations may promote carcinogenesis, whereas higher concentrations may inhibit tumorigenesis (Jaye et al., 2022b). Butyrate has demonstrated strong inhibitory effects on various breast cancer cell lines (Rodrigues et al., 2015; Semaan et al., 2020; Jaye et al., 2023). For example, sodium butyrate suppresses breast cancer cells by inducing cell cycle arrest at the G2/M phase, increasing caspase-10 levels, promoting apoptosis, and initiating intracellular calcium influx (Jaye et al., 2022a). Microbiota-derived butyrate can influence tumor progression by reshaping the TME. Butyrate’s interaction with its receptor Gpr109a exerts anti-inflammatory effects on colonic macrophages and dendritic cells, inducing regulatory T cell (Treg) differentiation and T cell production of IL-10, thereby suppressing colonic inflammation and cancer progression (Singh et al., 2014). Additionally, butyrate enhances ICB efficacy by modulating T cell receptor signaling in CD8+ T cells, indicating its potential as a therapeutic biomarker (Zhu et al., 2023). Despite butyrate’s considerable anti-tumor effects, low bioavailability and dose-dependent side effects have limited its clinical application. Nanoparticle-based delivery systems for butyrate may overcome these challenges (Yu et al., 2023).

#### 3.4.2 Bile acids

Most primary bile acids are reabsorbed in the small intestine, returned to the liver via the portal vein, and secreted back into the bile through enterohepatic circulation. Primary bile acids can also be metabolized by gut microbiota into secondary bile acids through deconjugation and dehydrogenation. Because breast cells do not produce bile acids, the presence of secondary bile acids in breast cancer tissues likely results from minimal leakage from the enterohepatic circulation or local production by the microbiome within the breast tissue. Wu et al. analyzed the transcriptomic and clinical case information of three large open primary breast cancer cohorts as well as the microbiome data of 16S rRNA gene sequences in TCGA breast cancer tissues. They found that breast tumors with low bile acid metabolism were more aggressive, and that there were a large number of microorganisms related to aggressive tumor biology in the TME. In breast tumors with high bile acid metabolism, oxidative stress-induced apoptosis leads to a significant increase in survival rate (Wu et al., 2022). This study indicates that bile acid metabolism usually inhibits tumor growth in breast cancer.

#### 3.4.3 Sodium deoxycholate

Sodium deoxycholate (DC), synthesized by intestinal bacteria, typically maintains a serum concentration of 5-10 μmol/L. However, in breast cyst fluid, DC levels can increase to more than 50 μmol/L. Although DC has been implicated in the promotion of colon carcinogenesis (Debruyne et al., 2002), its effects on breast cancer are complex.

DC plays a dual role in breast cancer epithelial cells, with lower concentrations promoting cell proliferation, likely through AKT phosphorylation and cyclin D1 expression, and higher concentrations inducing apoptosis and sustained activation of p38 and AKT (Gándola et al., 2020). This study suggests that the effects of bile salts on breast cancer cells are concentration-dependent. In a metastatic murine breast cancer model, DC act as natural tumor promoters by increasing Flk-1 and reducing ceramide-mediated progenitor cell apoptosis (Krishnamurthy et al., 2008).

Moreover, DC has been implicated in paclitaxel treatment-related peripheral neuropathy in patients with breast cancer. Paclitaxel treatment leads to the ingrowth of *Clostridium* species and increased DC levels. DC appear to elevate serum levels of CCL5/CCR5 in the dorsal root ganglion through the bile acid receptor TGR5, contributing to neuronal hyperexcitability and neuropathic pain (Zhong et al., 2023).

## 4 Intratumoral microbiota and breast cancer

Breast tissue was once considered a sterile environment. However, with an increasing number of studies focusing on the relationship between microorganisms and breast diseases, it has been found that breast tissue contains a variety of microbiota. The breast is primarily composed of adipose tissue with a rich vascular and lymphatic network, creating a conducive environment for bacterial growth, particularly of the phyla *Proteobacteria* and *Firmicutes*. *Streptococcus, Enterococcus*, and *Staphylococcus* can be isolated from both breast cancer and normal breast tissues, but the relative abundance at the genus level is very different, the expression of these microbiota in breast cancer tissue is significantly increased (Urbaniak et al., 2014, 2016; Thompson et al., 2017; Nejman et al., 2020; Fu et al., 2022). At the phylum level, *Proteobacteria* is predominant, followed by *Firmicutes*, *Bacteroidetes*, and *Actinobacteria*. The breast microbiome maintains healthy breast tissues by stimulating the resident immune cells (Xuan et al., 2014). Although the biomass of the intratumoral microbiota is very low, it plays an important role in promoting breast cancer progression.

### 4.1 Origin and colonization of breast cancer intratumoral microbiota

**Figure 1:**
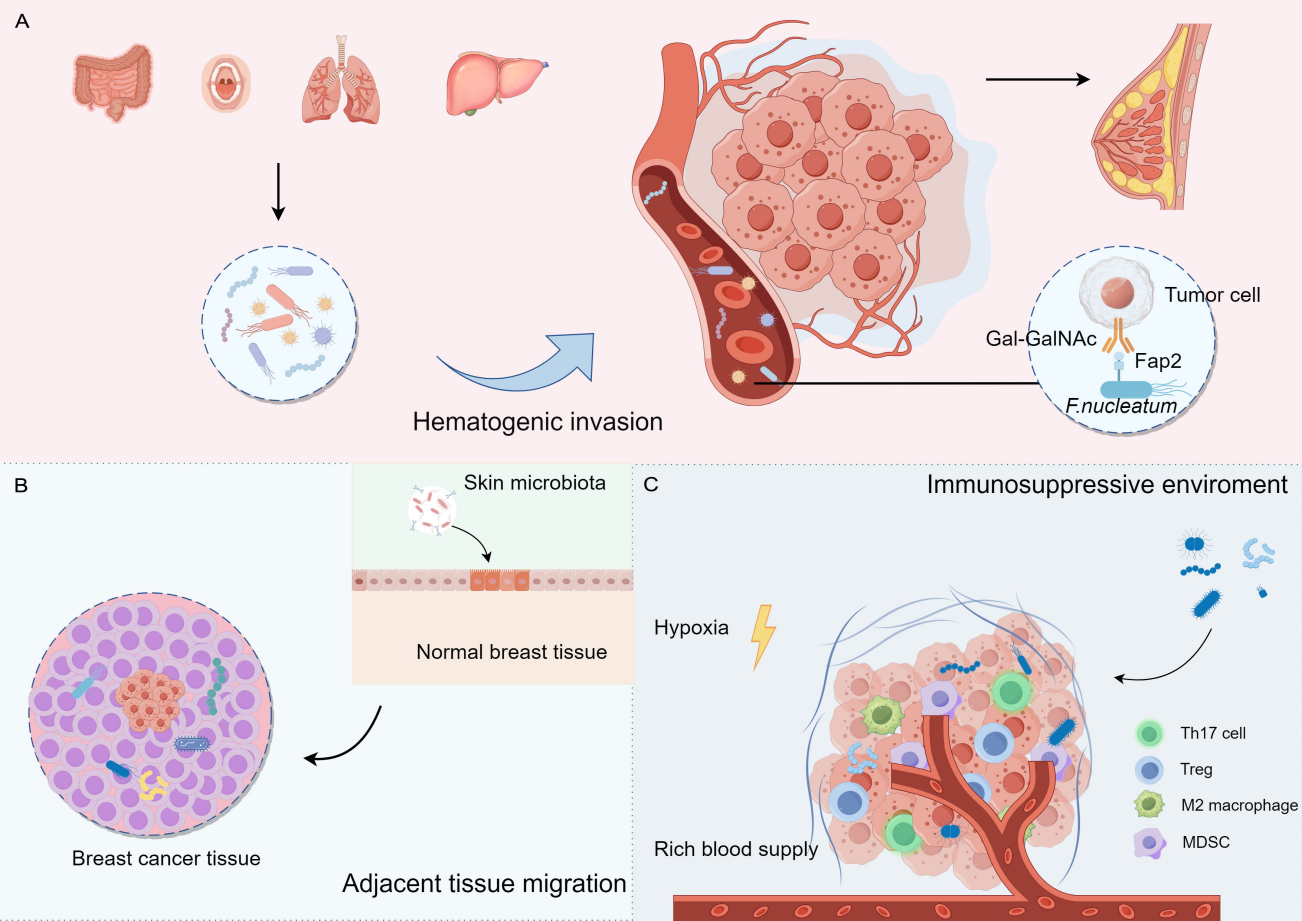
The potential origins and colonization of intratumoural microbiota in breast cancer. **(A)** Hematogenic invasion: Microorganisms from distant organs may reach breast tissue through the blood. **(B)** Adjacent tissue migration: Microorganisms from adjacent tissues may migrate to breast tissue (e.g., skin surface microorganisms). **(C)** Tumor microenvironment: tumor microenvironment (e.g., hypoxia, immunosuppression) may contribute to the colonization of microorganisms in the tumor tissue.

The origin and colonization of intratumoral microbiota remain uncertain. Microbiota may colonize tumor tissues through three primary mechanisms: mucosal destruction, hematogenic invasion, and adjacent tissue migration (Cao et al., 2024; Guo et al., 2024). Additionally, the immunosuppressive, hypoxic, and nutrient-rich environments in tumors may facilitate microbial colonization and reproduction (**Figure 1**).

*F. nucleatum* primarily presented in the oral cavity and gastrointestinal tract, has been implicated in various cancers, including breast cancer. This bacterium may promote tumorigenesis by affecting the infiltration and function of immune cells. Transient bacteremia during periodontal disease can facilitate oral *F. nucleatum* invasion into the bloodstream, leading to its translocation to the mammary glands. *F. nucleatum* can dock to tumor tissues via its surface-exposed lectin, Fap2, which recognizes the host Gal-GalNAc and enhances breast tumor growth and metastatic progression (Abed et al., 2016; Parhi et al., 2020). In addition, *Bacteroides* have been detected in canine breast tumors, as well as in the oral and gut microbiomes, suggesting a potential route of dissemination from the mouth to the gastrointestinal tract and ultimately to distant mammary tissue (Zheng et al., 2022). These studies indicate that oral microorganisms are a source of colonizing microorganisms in the breasts.

Adjacent normal tissues may also serve as potential sources of the intratumoral microbiota. The skin-related bacteria *Staphylococcus epidermidis* and *Micrococcus luteus* have been identified in mammary tumors, suggesting that these microbes may access the mammary duct through the nipple and disseminate to the mammary gland via the lobules and ducts (Urbaniak et al., 2014; Bernardo et al., 2023a). The similarities between the microbiome communities in tumors and adjacent normal tissues support the possibility of adjacent tissue invasion.

### 4.2 Differences between the microbiota in normal breast and breast cancer tissues

*Methylobacterium radiotolerans*, *Bacillus, Enterobacteriaceae, Staphylococcus, Ralstonia, Bacillaceae*, and *Burkholderiaceae* are more abundant in breast cancer and their adjacent tissues, whereas *Sphingomonas yanoikuyae, Acetobacter aceti, Lactobacillus vini*, and *Lactobacillus paracasei* are more abundant in healthy breast tissues (Xuan et al., 2014; Urbaniak et al., 2016; Hoskinson et al., 2022; German et al., 2023). Analysis of microbiome-immune networks in the breast has revealed that *Anaerococcus*, *Caulobacter*, and *Streptococcus* are crucial hubs in benign tissues but are absent in tumor tissues (Tzeng et al., 2021). Taken together, these studies indicate that both healthy breast tissues and breast cancer tissues have unique microbial environments.

The intratumoral microbiota of patients with breast cancer also varies according to race and sex. Metagenomic analyses have identified race-associated microbial biomarkers, such as *Pseudomonas* and *Methylobacter* in tumors from Asian women, and *Amycolatopsis* in tumors from black women (Siddharth et al., 2021; Parida et al., 2023b). Notably, *Ralstonia* was most enriched in non-Hispanic Black patients, whereas *Xanthomonadales* were more prevalent in non-Hispanic white patients (Smith et al., 2019). Differences in the breast microbiomes of men and women have also been reported. *Tenericutes*, particularly *Mesoplasma* and *Mycobacterium*, are implicated in breast carcinogenesis in both sexes (Niccolai et al., 2023). Dysbiosis extends throughout the breast tissue in females and is more localized to the tumor site in males.

Microbial signatures differ among breast cancer subtypes. Actinomyces signatures have been detected across all breast cancer types, with higher signal intensities observed in patients with HER2+ breast cancer (Banerjee et al., 2018). A larger cohort analysis revealed that each subtype had unique microbial signatures, with ER+ breast cancer showing the most diverse tumor microbiome and TNBC exhibiting the least diversity. Notably, higher abundances of *Bacillus*, *Mucor*, *Nodaviridae*, *Toxocara*, and *Trichophyton* in TNBC samples were significantly correlated with a better prognosis (Banerjee et al., 2021).

### 4.3 Role of intratumoral microbiota in breast cancer development

**Figure 2:**
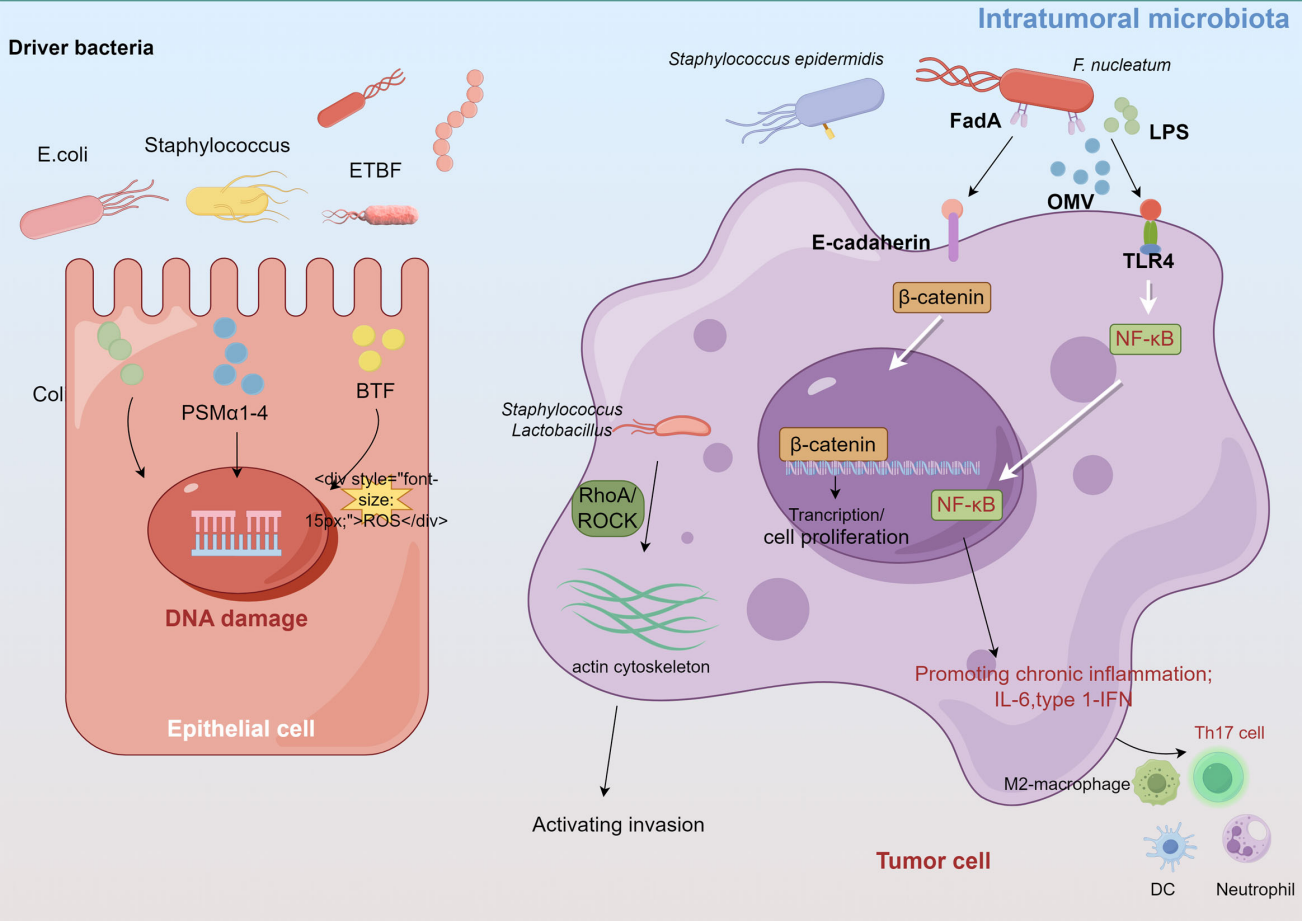
Intratumoral microbiota affect tumor development through several proposed mechanisms, such as DNA damage, promoting immune and inflammation, activating invasion and metastasis.

Although the direct effects of the intratumoral microbiota on breast cancer have been less frequently reported, evidence suggests that the intratumoral microbiota may regulate the development of breast cancer through inducing genomic instability and DNA mutations, activating carcinogenic pathways, promoting inflammatory responses, and modulating the local immune microenvironment to facilitate invasion (Xie et al., 2022; Yang et al., 2023a; Cao et al., 2024) (**Figure 2**).

#### 4.3.1 Induce DNA damage

Certain carcinogenic bacteria, such as pks+ *Escherichia coli* and *Staphylococcus aureus*, encode and secrete cytolytic toxins (e.g., colibactin, PSMα1-4) that cause ROS-mediated DNA damage and accelerate tumor onset (Dejea et al., 2018; Krueger et al., 2022). These bacteria, which belong to the Enterobacteriaceae and *Staphylococcus* genera, are enriched in breast cancer tissues. Guo et al. found that *F. nucleatum*, abundant in the human breast cancer microbiome, secretes adhesin FadA to activate the E-cadherin/β-catenin pathway, upregulate Chk2 levels and induce DNA damage (Guo et al., 2020a). Moreover, the gut-colonizing bacterium *enterotoxigenic Bacteroides fragilis* (ETBF) in the mammary gland increases the expression of spermine oxidase in intestinal epithelial cells, leading to ROS production and γ-H2A activation that causes DNA damage (Goodwin et al., 2011; Parida et al., 2021).

#### 4.3.2 Regulation of the inflammatory response

Dysregulated innate immunity in cancer often causes persistent chronic inflammation, which promotes cancer progression and anti-tumor immune resistance. Intratumoral microbiomes can activate inflammatory signals by interacting with pattern recognition receptors (PRRs) such as TLRs in the TME. The intratumoral microbiota in breast cancer significantly influence TLR signaling, particularly through the LPS/TLR4 pathway cascade (Afroz et al., 2022; Wilkie et al., 2022). *F. nucleatum* and *A. actinomycetemcomitans* also activate diverse TLRs and NF-κB in bone marrow-derived macrophages, increasing IL-6 production (Park et al., 2014). Untreated murine mammary tumors display increased *Staphylococcus* abundance, of which isolated *Staphylococcus epidermidis* demonstrates significant inflammatory activity (Bernardo et al., 2023b). Moreover, bioinformatics analysis revealed that *Propionibacterium* and *Staphylococcus*, which are decreased in tumors, correlate negatively with oncogenic immune signatures, whereas *Streptococcus* and *Propionibacterium* correlate positively with T cell activation (Tzeng et al., 2021). Mammary metabolism-related microbiota are related to T cell exclusion and immunotherapy responses (Chen et al., 2023).

#### 4.3.3 Promoting invasion and metastasis

Intratumoral microorganisms influence both intercellular interactions and the external microenvironment, facilitating distant metastasis (Cao et al., 2024). Fu et al. demonstrated that in mouse models of spontaneous breast tumors (MMTV-PyMT), the intratumoral bacteria, carried by circulating tumor cells, can modulate RhoA/ROCK signaling pathways, alternate the actin cytoskeleton, enhance resistance of host cells to fluid shear stress, and promote lung metastasis in breast cancer, thereby promoting the survival and metastasis of host cells (Fu et al., 2022).

The colonic oncogenic microorganism ETBF, found in carcinogenic breast tissues, secretes a toxin that induces hyperplasia in breast epithelial cells (Parida et al., 2021). ETBF is not present in normal breast tissues. Compared with non-toxigenic *Bacteroides fragilis*, ETBF colonizing in the breast and intestinal ducts can affect epithelial-mesenchymal transition (EMT) by activating the β-catenin and Notch1 signaling pathways, significantly promoting tumor growth and metastasis. ETBF infection can also trigger systemic inflammation in breast cancer, increasing the levels of proinflammatory and tumorigenic cytokines such as IL-17A and IL-6 (Parida et al., 2023a). These inflammatory changes reshape the TME, create a pre-metastatic niche in the target organs, and promote metastasis to the lungs and liver. *F. nucleatum*-derived outer membrane vesicles also promote lung metastasis in tumor-bearing mice by altering EMT-related protein levels and activating intracellular autophagy pathways (Chen et al., 2024a). Small extracellular vesicles derived from *F. nucleatum* in breast cancer facilitate tumor growth and metastasis via TLR4 signaling (Li et al., 2023). Thus, extracellular vehicles play a role in modulating communication between cancer cells and the surrounding microenvironment and distant organs.

### 4.4 Influence of intratumoral microorganisms on the chemotherapy of breast cancer

A study by Gao et al. demonstrated that *F. nucleatum* can induce PD-L1 expression through activating STING signals, leading to accumulation of IFN-γ+ CD8+ lymphocytes (Gao et al., 2021). This could potentially improve the efficacy of ICBs in the treatment of CRC. Similarly, the pathogenic bacterium *Salmonella typhimurium* exerts potent anti-tumor effects by activating the immune system (Zheng et al., 2017; Guo et al., 2020b). These studies indicate that symbiotic microorganisms play important roles in regulating host immune responses and therapeutic effects.

Tumor-resident intracellular microbiome (TRIM) can enhance tumor cell proliferation and metastatic colonization while decreasing chemotherapy efficacy (Sears et al., 2014; Geller et al., 2017; Fu et al., 2022). ETBF-secreted toxins enhance cancer cell stemness and chemoresistance by activating NUMB phosphorylation, which leads to lysosomal degradation and Notch1 activation (Ma et al., 2024). In CRC, *F. nucleatum* induces resistance to oxaliplatin and 5-fluorouracil (5-FU) by upregulating autophagy through TLR4/MYD88-dependent signals and preventing apoptosis via the upregulation of ANO1/BIRC3 (Yu et al., 2017; Zhang et al., 2019). In addition, *F. nucleatum* is involved in promoting chemotherapy resistance in esophageal squamous cell carcinoma (Liang et al., 2022) and oral squamous cell carcinoma (Liu et al., 2021a). In an animal model of breast cancer, *F. nucleatum* colonization was linked to reduced chemotherapy efficacy by activating autophagy-related pathways in cancer cells (Su et al., 2024).

Combining targeted antibacterial treatments with chemotherapy can suppress both TRIM and tumor cells, promoting M2-to-M1-like tumor-associated macrophage repolarization and achieving long-term survival in animal models with no recurrence (Wang et al., 2024). *F. nucleatum*-mimicking nanovehicles, which fuse cytoplasmic membranes with antibiotic-loaded liposomes, have been shown to selectively target and eradicate tumor-resident bacteria, thereby significantly restoring the effectiveness of chemotherapy (Chen et al., 2024c). The biomimetic nanocarriers improve the immunosuppressive TME induced by intratumoral *F. nucleatum* and enhance the therapeutic effect of PD-L1 (Geng et al., 2024). These studies indicate that targeting pathogenic microorganisms in tumors may help improve the efficacy of chemotherapy and prevent recurrence.

Additionally, nanoparticles coated with bacteria-derived outer membrane vesicles (OMVs) convert intratumor *F. nucleatum* into immunopotentiators, releasing pathogen-associated molecular patterns (PAMPs) and enhancing immunochemodynamic therapy efficacy in TNBC (Liu et al., 2023). Encapsulating bacteria-derived extracellular vesicles (BEVs) in nanocloaks can increase immunogenicity and facilitate DC maturation by activating cGAS-STING signaling. This approach, in combination with anti-PD-L1 antibodies, elicits a potent immune response and synergistically inhibits tumor progression and lung metastasis (Zhang et al., 2024). Therefore, bacteria-derived vesicles can effectively improve the immunosuppressive state of the TME, and represent a potential treatment method.

### 4.5 Prognostic value of microbiomes and miRNA

Intratumoral microbiome profiles may serve as valuable tools for predicting patient prognosis and assessing the clinical efficacy of specific drugs. A recent study by Li et al. identified four immune-related intratumor microbiomes (IRIM; e.g., *Acidibacillus* and *Succinimonas*) that have potential prognostic value (Li J. et al., 2024). These microbiomes were correlated with immune gene levels and sensitivity to chemotherapeutic agents, particularly tamoxifen and docetaxel.

Microbiota dysbiosis can trigger various immune-mediated diseases by regulating the derived metabolites and host environmental factors. miRNAs have emerged as critical mediators of host-microbiome interactions, with bidirectional effects observed between the microbiome and miRNAs in carcinogenesis (Yang et al., 2017; Yuan et al., 2019; Wang et al., 2021).Yang et al. Found that patients with both high amount of tissue *Fusobacterium nucleatum* DNA and miR21 had a higher risk of poor prognosis (Yang et al., 2017). Laborda-Illanes et al. revealed an increase in the expression of miR-149-5p, miR-20b-5p, and miR-342-5p in metastatic breast cancer (Met-BC) patients, compare with non-metastatic breast cancer (nonMet-BC) patients (Laborda-Illanes et al., 2024). The Met-BC group exhibited an increase in several pathogenic and pro-inflammatory species, including *Streptococcus epidermidis, Haemophilus influenzae, Corynebacterium aurimucosum, and Corynebacterium kroppenstedtii*, while the NonMet-BC group displayed higher levels of probiotic bacteria, such as *Parabacteroides distasonis, Lactobacillus iners, Blautia obeum, and Faecalibacterium prausnitzii* (Laborda-Illanes et al., 2024). These studies suggest that consideration of both intratumoral miRNA expression and microbiota changes could aid in precision treatment for breast cancer.

## 5 Future outlook and conclusion

In summary, the influence of the gut and local microbiota on breast cancer development requires further investigation to elucidate the underlying mechanisms and identify key microbial players (Bernardo et al., 2023a). Challenges such as low microbial biomass, environmental contamination from non-microbial genomes, and antibiotic perturbations persist. Additionally, animal models used in bacterial research may not fully represent the human microbiome owing to differences in diet, genetics, and age. Co-culturing specific bacterial taxa (e.g., *Streptococcus, Lactobacillus, Salmonella, Bifidobacterium*) with pluripotent stem cells or organoids may be a promising approach for studying host-microbe interactions (Puschhof et al., 2021; Kim et al., 2022; Morelli et al., 2023). Regarding microbiota-related metabolites, patient-derived tumor spheroids could provide insights into the potential therapeutic use of these metabolites; however, effective therapeutic doses need to be determined. Beneficial bacterial or microbiota-based therapies may enhance hormonal, metabolic, and immune regulation in hormone receptor-positive cancers. Strategies such as the administration of prebiotics, probiotics, and fecal FMT could be beneficial (Zitvogel et al., 2008; Terrisse et al., 2023). Microbes that promote tumors can be eradicated using antibiotics or phage therapies.

Future research involving human subjects is crucial to unravel the complex interactions among the microbiome, disease, and the host. The goal is to explore new therapeutic avenues that modulate the gut and/or local microbiota to create a more favorable TME (Fessler et al., 2019; Laborda-Illanes et al., 2020). Personalized assessment of a patient’s gut and tumor microbiome composition could serve as a diagnostic and prognostic tool, potentially improving treatment outcomes and patient prognosis.
